# Survival of the Fittest: The Relationship of (p)ppGpp With Bacterial Virulence

**DOI:** 10.3389/fmicb.2020.601417

**Published:** 2020-12-03

**Authors:** Shivani Kundra, Cristina Colomer-Winter, José A. Lemos

**Affiliations:** Department of Oral Biology, UF College of Dentistry, Gainesville, FL, United States

**Keywords:** (p)ppGpp, stringent response (SR), bacterial virulence, bacterial stress response, regulatory nucleotides

## Abstract

The signaling nucleotide (p)ppGpp has been the subject of intense research in the past two decades. Initially discovered as the effector molecule of the stringent response, a bacterial stress response that reprograms cell physiology during amino acid starvation, follow-up studies indicated that many effects of (p)ppGpp on cell physiology occur at levels that are lower than those needed to fully activate the stringent response, and that the repertoire of enzymes involved in (p)ppGpp metabolism is more diverse than initially thought. Of particular interest, (p)ppGpp regulation has been consistently linked to bacterial persistence and virulence, such that the scientific pursuit to discover molecules that interfere with (p)ppGpp signaling as a way to develop new antimicrobials has grown substantially in recent years. Here, we highlight contemporary studies that have further supported the intimate relationship of (p)ppGpp with bacterial virulence and studies that provided new insights into the different mechanisms by which (p)ppGpp modulates bacterial virulence.

## Introduction

**FIGURE 1:**
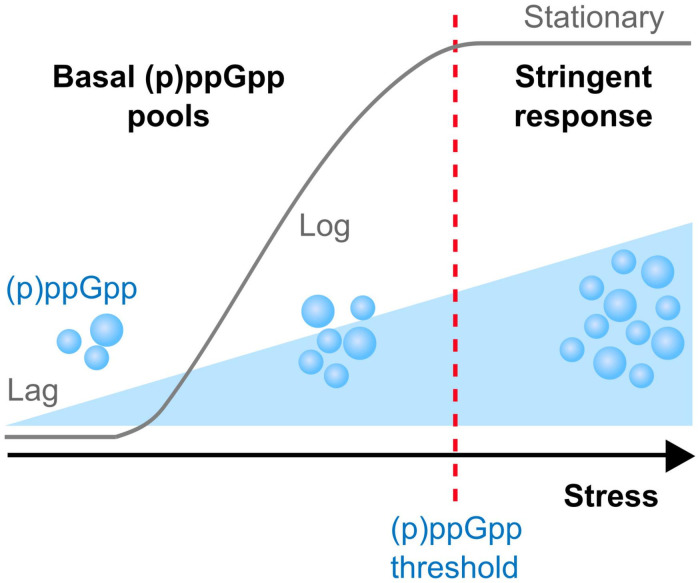
(p)ppGpp modulates bacterial physiology at all growth stages. During adaptation (lag) and exponential (log) growth phases in the absence of stresses, basal levels of (p)ppGpp are important to maintain a balanced metabolism avoiding uncontrolled consumption of energy stores and toxic accumulation of metabolic byproducts. Entry into stationary growth phase or adverse condtions such as nutrient starvation, heat shock or selected antibiotic stresses triggers the accumulation of (p)ppGpp that rapidly reaches the threshold necessary to mount the SR responsible for remodeling cell physiology from a growth mode to a survival mode.

**FIGURE 2:**
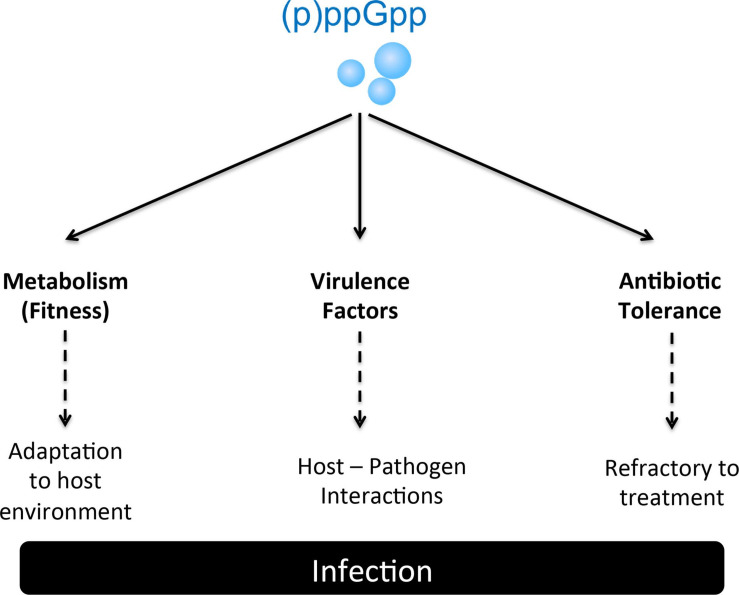
(p)ppGpp participates in the regulation of several processes critical to bacterial pathogenesis. The roles of (p)ppGpp in adaptation to the host environment and antibioric tolerance are likely universal whereas the association with the expression of virulence factors appears to be species-specific.

In response to changes in the surrounding environment, bacteria utilize a variety of sophisticated sensory mechanisms that reprogram cell physiology to facilitate adaptation to the new environment. Among those mechanisms are the production of signaling nucleotides such as (i) cAMP, the first regulatory nucleotide ever described, (ii) a growing family of cyclic nucleotides such as c-di-GMP, c-di-AMP, and cAMP-GMP, and (iii) hyperphosphorylated nucleotides, including (p)ppGpp and newly described analogs pGpp and (p)ppApp (Potrykus and Cashel, 2008; Kalia et al., 2013; Gaca et al., 2015b; Yang et al., 2019, 2020; Fung et al., 2020; Jimmy et al., 2020). The commonly used (p)ppGpp abbreviation indicates two guanosine derivatives – ppGpp (GDP, 3′-diphosphate) and pppGpp (GTP, 3′-diphosphate) – initially known as the magic spot or nutritional alarmone (Cashel and Kalbacher, 1970; Potrykus and Cashel, 2008). Seminal studies discovered that in response to stress conditions, (p)ppGpp rapidly accumulates to high levels within the cell and reprograms cell physiology through transcriptional and allosteric mechanisms that ultimately reallocate cellular resources from an active growth state toward a semi-dormant state (Potrykus and Cashel, 2008). When fully active, this process termed the stringent response (SR), has been shown to lower the activity of metabolic pathways associated with rapid cell growth, while activating pathways associated with nutrient uptake, amino acid biosynthesis and stress survival thereby facilitating cell survival under severe adverse conditions (Potrykus and Cashel, 2008; Kanjee et al., 2012; Gaca et al., 2015a). While initially discovered as a response to amino acid starvation, the SR was also shown to be induced by non-nutritional stresses such as heat stress and antibiotics (Gallant et al., 1977; Glass et al., 1979; Wells and Gaynor, 2006; Hobbs and Boraston, 2019; Schafer et al., 2020). In addition to acting as the effector molecule of the SR, contemporary studies revealed that (p)ppGpp plays a fundamental role in the control of core cellular processes at concentrations that are well below those needed to activate the SR (Figure 1). Indeed, during balanced (non-stressed) conditions, relatively small fluctuations in basal (p)ppGpp pools were shown to influence transcription of hundreds of genes, with a complete loss of (p)ppGpp regulation impairing cell fitness even in the absence of stress (Gaca et al., 2013; Colomer-Winter et al., 2019; Sanchez-Vazquez et al., 2019; Fernandez-Coll et al., 2020; Pletzer et al., 2020). Given the critical role of (p)ppGpp in cell physiology, it is not surprising that several studies have implicated (p)ppGpp with bacterial virulence and antibiotic tolerance (Table 1 and Figure 2). Specifically, the production of (p)ppGpp has been associated with expression of virulence traits, which includes but is not limited to adhesion, biofilm formation, toxin production, motility, sporulation, and antibiotic tolerance (Dalebroux et al., 2010). Importantly, several of those studies indicate that the intimate relationship of (p)ppGpp with bacterial persistence and virulence might be more closely linked to basal levels of (p)ppGpp than to those needed to activate the SR. In 2010, Dalebroux and colleagues published a comprehensive review linking (p)ppGpp to bacterial virulence in plants, animals and humans (Dalebroux et al., 2010). Here, we will highlight recent studies that have further supported the intimate relationship of (p)ppGpp with bacterial virulence, with an emphasis on studies that directly tested (p)ppGpp-deficient strains in animal infection models that are relevant to human infectious diseases. For historical and contemporary perspectives of other aspects of the field, we direct the reader to other reviews (Dalebroux and Swanson, 2012; Gaca et al., 2015a; Hauryliuk et al., 2015; Liu et al., 2015; Steinchen and Bange, 2016; Ronneau and Hallez, 2019; Zhu et al., 2019b).

**TABLE 1:** Virulence of (p)ppGpp-defective mutants.

Bacterial Pathogen	Strain	Basal (p)ppGpp	Stringent response	Animal Model/Virulence	References
*Acinetobacter baumanii*	ΔrelA	Yes	No	*G. mellonella*: attenuated	Perez-Varela et al. (2020)
*Bacillus anthracis*	Δrel	Yes	No	Mouse subcutaneous: ND1	van Schaik et al. (2007)
*Borrelia burgdorferi*	Δrel	No	No	Mouse intradermal: avirulent	Bugrysheva et al. (2005)
*Brucella melitensi*s	Δrel	No	No	Mouse systemic: attenuated	Dozot et al. (2006)
*Burkholderia pseudomallei*	ΔrelAΔspoT	No	No	*G. mellonella* and mouse: attenuated	Muller et al. (2012)
*Enterococcus faecalis*	Δrel	Yes	No	*G. mellonella* or *C. elegans*: ND; Mouse CAUTI: ND; Rabbit abscess: ND; Rabbit endocarditis: attenuated	Abranches et al. (2009); Yan et al. (2009), Gaca et al. (2012); Frank et al. (2014), Colomer-Winter et al. (2018, 2019)
	ΔrelQ	Yes	Yes	*G. mellonella* or *C. elegans*: ND; Mouse CAUTI: ND; Rabbit abscess: ND; Rabbit endocarditis: ND	Abranches et al. (2009); Gaca et al. (2012), Frank et al. (2014); Colomer-Winter et al. (2018)Colomer-Winter et al. (2019)
	ΔrelΔrelQ	No	No	*G. mellonella* or *C. elegans*: attenuated; Mouse CAUTI: attenuated; Rabbit abscess: attenuated; Rabbit endocarditis: ND	Abranches et al. (2009); Gaca et al. (2012), Frank et al. (2014); Colomer-Winter et al. (2018)Colomer-Winter et al. (2019)
*Francisella novocida*	ΔrelA	Yes	No	Mouse (intranasal): attenuated	Dean et al. (2009)
*Francisella tularensis*	ΔrelAΔspoT	No	No	Mouse (intranasal): avirulent	Charity et al. (2009); Ma et al. (2019)
*Haemophilus ducreyi*	ΔrelAΔspoT	No	No	Human pustule: attenuated	Holley et al. (2014)
*Listeria monocytogenes*	Δrel	Yes	No	Mouse systemic: avirulent	Taylor et al. (2002), Bennett et al. (2007)
	rel:Tn	?	?	Mouse systemic: attenuated	Taylor et al. (2002)
	ΔrelΔrelPΔrelQ	No	No	Mouse systemic: attenuated	Whiteley et al. (2015)
*Mycobacterium tuberculosis*	relH344Y	Yes	No	Mouse lung: attenuated	Weiss and Stallings (2013)
	relH80A	Yes	Yes	Mouse lung: attenuated	Weiss and Stallings (2013)
	ΔrelΔSAS	No	No	Mouse lung: attenuated	Weiss and Stallings (2013)
	Δrel	Yes	No	Mouse lung and guinea pig lung: attenuated	Dahl et al. (2003); Klinkenberg et al. (2010)
*Pseudomonas aeruginosa*	ΔrelA	Yes	No	*D. melanogaster*: attenuated; Mouse abscess: ND	Vogt et al. (2011); Pletzer et al. (2017)
	ΔrelAΔspoT	No	No	*D. melanogaster*, Mouse pneumonia: avirulent/attenuated; Mouse abscess/skin: attenuated	Vogt et al. (2011); Xu et al. (2016), Pletzer et al. (2017)
*Salmonella Gallinarum*	ΔrelAΔspoT	No	No	Chicken (oral): attenuated	Park et al. (2010)
*Salmonella Typhi*	ΔrelAΔspoT	No	No	Mouse (oral): avirulent	Dasgupta et al. (2019)
*Salmonella Typhimurium*	ΔrelA	Yes	No	Mouse (intragastric): attenuated	Pizarro-Cerda and Tedin (2004)
	spoT-Δctd	Yes	Yes	Mouse (oral): attenuated	Fitzsimmons et al. (2020)
	ΔrelAΔspoT	No	No	Mouse (intragastric): avirulent	Pizarro-Cerda and Tedin (2004)
*Staphylococcus aureus*	relSaF128Y	High	Yes	*G. mellonella*: attenuated	Gao et al. (2010)
	relSasyn	Yes	No	Mouse kidney: attenuated; Mouse skin: attenuated	Geiger et al. (2010); Mansour et al. (2016)
	ΔrelP	Yes	Yes	Rabbit endocarditis: ND	Li et al. (2020)
*Streptococcus pneumoniae*	ΔrelSpn	Yes	No	Mouse pneumonia: attenuated	Kazmierczak et al. (2009)
*Streptococcus suis*	ΔrelΔrelQ	No	No	Mouse systemic: avirulent	Zhu et al. (2016)
*Vibrio cholerae*	ΔrelA	Yes	No	Suckling mouse: attenuated/ND 2; Rabbit ileal loop: attenuated	Haralalka et al. (2003)
	ΔrelAΔspoT	Yes	No	Infant mouse: ND	Oh et al. (2014)
	ΔrelAΔspoTΔrelV	No	No	Infant mouse: attenuated	Oh et al. (2014)
*Yersinia pestis*	ΔrelA	Yes	No	Mouse: ND	Sun et al. (2009)
	ΔrelAΔspoT	No	Yes	Mouse: attenuated	Sun et al. (2009)

*ND, no differences when compared to parent strain. Conflicting data from different laboratories.*

## Gram-Positive Pathogens

Most of the Gram-positive bacteria that cause disease in humans belong to the Firmicutes phylum, which is primarily comprised of bacteria with a low-GC content. In Firmicutes, (p)ppGpp metabolism is primarily controlled by the bifunctional synthetase/hydrolase Rel enzyme, also known as RelA or Rsh (for RelA SpoT homolog) (Atkinson et al., 2011). The use of different designations for the (p)ppGpp synthetase/hydrolase of Gram-positive bacteria has created confusion in the field, since the Gram-negative RelA protein (originally described in *Escherichia coli*) is a monofunctional synthetase without hydrolase activity. Though some studies might have originally referred to the bifunctional (p)ppGpp synthetase/hydrolase of Gram-positive bacteria as RelA or Rsh, herein, we will adopt the nomenclature proposed by Atkinson and colleagues (Atkinson et al., 2011) and refer to the bifunctional Gram-positive enzyme as Rel.

While Rel-dependent (p)ppGpp synthesis is primarily responsible for activation of the SR in all Gram-positive bacteria investigated to date, the (p)ppGpp hydrolase activity of this enzyme is essential to some species by avoiding the accumulation of (p)ppGpp to toxic levels (Geiger et al., 2010; Weiss and Stallings, 2013; Gratani et al., 2018; Ronneau and Hallez, 2019). In addition to Rel, Firmicutes express one or two short/small (p)ppGpp synthetases (SASs), termed RelP and RelQ (known as YjbM and YwaC, respectively, in *Bacillus subtilis*) (Atkinson et al., 2011). All SASs appear to be primarily involved in maintainence of basal (p)ppGpp pools during growth, though have been also linked to a timely activation of the SR (Gaca et al., 2012) and to cell envelope stress tolerance (Geiger et al., 2014; Bhawini et al., 2019). While the majority of Firmicutes encode both RelP and RelQ, few species including *Streptococcus pneumoniae*, *Streptococcus suis* and all members of the *Enterococcus* genus only encode RelQ (Atkinson et al., 2011). Below, we review studies that, either through the use of animal models, clinical evidence, or both, provided conclusive evidence of the association of (p)ppGpp with Gram-positive bacterial virulence. When discussing these studies it is important to mention that different than Gram-negative bacteria, (p)ppGpp does not control global gene transcription by physically interacting with the RNAP and by partnering with the transcriptional regulator DksA (Wolz et al., 2010). Instead, (p)ppGpp indirectly affects Gram-positive gene transcription through modulation of intracellular purine concentrations thereby changing the availability of initiating nucleotides of transcription (Krasny and Gourse, 2004; Krasny et al., 2008; Kriel et al., 2012). In addition, the sharp drop in GTP and concomitant increase in ATP directly affects activity of CodY, a nutrient-sensing transcriptional regulator that is widespread in Firmicutes and that controls activation of nutrient-acquisition and nutrient biosynthesis pathways and virulence determinants (Sonenshein, 2005). Because CodY is a GTP-responsive regulator, there is an inverse relationship between (p)ppGpp and CodY where the accumulation of (p)ppGpp typically leads to alleviation of CodY regulation (Geiger and Wolz, 2014). As a result, the (p)ppGpp and CodY networks are intertwined, with several reports linking both regulatory networks to bacterial virulence and, in most cases, having an antagonistic relationship (Geiger and Wolz, 2014). In addition to CodY, (p)ppGpp control of bacterial physiology is also exherted through direct interaction with enzymes and riboswitches (Kanjee et al., 2012; Sherlock et al., 2018).

### Staphylococcus aureus

A common inhabitant of the human upper respiratory tract, *S. aureus* is also a major threat to public health as they can cause a wide range of opportunistic infections that range from mild skin infections to life-threatening pneumonia, osteomyelitis, endocarditis and sepsis. In *S. aureus*, (p)ppGpp is metabolized by the bifunctional Rel_*Sa*_ (Rsh_*Sa*_) and the monofucntional RelP and RelQ. While the Rel_*Sa*_ synthetase was dispensable, its hydrolase activity was found to be essential in the presence of functional RelP and RelQ by preventing toxic accumulation of (p)ppGpp due to the activities of SASs (Geiger et al., 2010). The contribution of (p)ppGpp and the SR to staphylococcal virulence was first demonstrated in a synthetase-dead/hydrolase-active Rel mutant strain (*rel*_*syn*_), where loss of Rel_*Sa*_ synthetase activity significantly attenuated *S. aureus* virulence in a mouse model of kidney infection (Geiger et al., 2010). Follow-up studies testing this mutant confirmed that Rel-dependent (p)ppGpp accumulation was also required for lesion formation in a cutaneous mouse model (Mansour et al., 2016). Notably, virulence of the *rel*_*syn*_ mutant was restored by deletion of *codY*, providing clear evidence of the (p)ppGpp-CodY relationship during infection (Geiger et al., 2010). Subsequent studies revealed that most genes activated by (p)ppGpp in *S. aureus* were under CodY control (Geiger et al., 2012), indicating that activation of the SR (i.e., high levels of (p)ppGpp) promotes virulence by, at least in part, alleviation of CodY regulation (Geiger et al., 2010, 2012). In addition to virulence, the SR has been associated to antibiotic tolerance (often referred as antibiotic persistence). For instance, a drug-resistant clinical isolate obtained from a persistent bloodstream infection treated with the protein synthesis inhibitor linezolid was shown to harbor point mutations in the *rel*_*Sa*_ gene that led to constitutive activation of the SR (Gao et al., 2010).

In addition to activation of the SR, more recent studies indicated that basal levels of (p)ppGpp (below those needed to activate the SR) contribute to antibiotic tolerance. Specifically, point mutations on the *rel*_*Sa*_ gene lowered the (p)ppGpp hydrolase activity of Rel_*Sa*_ causing a slight increase in basal (p)ppGpp pools that was linked to broad antibiotic tolerance (Mwangi et al., 2013; Bryson et al., 2020). In another study, the *relP*_*Sa*_ and *relQ*_*Sa*_ genes were shown to be strongly induced upon treatment with cell wall-active antibiotics (ampicillin and vancomycin) and simultaneous inactivation of both genes significantly decreased tolerance of *S. aureus* toward these antibiotics (Geiger et al., 2014). In a more recent study, the association of SASs with antibiotic tolerance was traced to RelQ as inactivation of *relQ*_*Sa*_ decreased tolerance to β-lactam antibiotics in methicillin-resistant *S. aureus* (MRSA) by interfering with *mecA* gene expression, which codes for an alternative penicillin-binding protein (Bhawini et al., 2019). Interestingly, the importance of RelQ_*Sa*_ in β-lactam tolerance could be bypassed by activation of the SR with mupirocin (an isoleucine analog/^*ile*^tRNA inhibitor that triggers the SR), suggesting that *mecA* transcription requires a certain (p)ppGpp threshold (Bhawini et al., 2019). Unexpectedly, single inactivation of *relP*_*Sa*_ increased β-lactam resistance, which the authors attributed to a sizeable increase in the expression of the *relQ*_*Sa*_ promoter in the Δ*relP* strain (Bhawini et al., 2019). MRSA isolates from a clinical case with high tolerance to vancomycin displayed high levels of (p)ppGpp, high expression of cytotoxic PSMs (phenol-soluble modulins), increased PMN lysis and intracellular survival, and enhanced adherence to fibronectin/endothelial cells in *in vitro* assays (Li et al., 2020). Interestingly, transcription activation of PSMs was found to be both (p)ppGpp- and CodY-independent (Geiger et al., 2012). Finally, virulence of the *relP* mutant was not affected in a rabbit infective endocarditis (IE) model but treatment of the heart vegetation colonized by the *relP* mutant with vancomycin reduced spread in the cardiac vegetation and dissemination to other tissues (Li et al., 2020). Collectively, these studies reveal that the SR and CodY mediate *S. aureus* virulence and antibiotic tolerance, but also indicate that basal levels of (p)ppGpp, primarily mediated by RelP and RelQ, play a role in supporting staphylococcal cell attachment, survival to immune cells and antibiotic tolerance. In the future, it will be important to determine the relevance of (p)ppGpp and the SR in other types of infection and evaluate the virulence potential of a triple Δ*rel*Δ*relP*Δ*relQ* mutant [(p)ppGpp^0^ strain].

### Enterococci

A natural inhabitant of the gastrointestinal tract of animals, enterococci are among the leading causes of life-threatening nosocomial infections (Arias and Murray, 2012). While *E. faecalis* accounts for the majority of enterococcal infections in humans (∼75%), *E. faecium* responds for the majority of vancomycin resistant enterococci (VRE) infections. The genome of *E. faecalis* encodes a bifunctional Rel (Rel_*Ef*_) and a single SAS termed RelQ_*Ef*_. Similar to *S. aureus*, Rel_*Ef*_ is the major enzyme responsible for (p)ppGpp accumulation and activation of the SR (Abranches et al., 2009; Gaca et al., 2012), an observation that has been confirmed with all other Gram-positive bacteria that have been studied to date. In the past decade, the importance of (p)ppGpp and the SR to *E. faecalis* pathophysiology has been probed to some detail by our group. Collectively, our studies indicate that the association of (p)ppGpp with virulence does not necessarily relate to activation of the SR but to basal levels of (p)ppGpp (Abranches et al., 2009; Gaca et al., 2012, 2013). Specifically, we and others showed that the consequences of single inactivation of *rel*_*Ef*_ or *relQ*_*Ef*_ to *E. faecalis* pathophysiology are for the most part negligible or fairly modest (Yan et al., 2009; Frank et al., 2014; Colomer-Winter et al., 2018, 2019) whereas a Δ*rel*Δ*relQ* double mutant [(p)ppGpp^0^] strain showed multiple phenotypes including impaired survival within the macrophage cell line J774.A1, growth/survival defects in human fluids (blood and urine) *ex vivo*, and attenuated virulence in invertebrate (*Caenorhabditis elegans* and *Galleria mellonella*) and vertebrate [rabbit subdermal abscess and mouse (CAUTI) catheter-associated urinary tract infection] models (Abranches et al., 2009; Gaca et al., 2012; Frank et al., 2014; Colomer-Winter et al., 2019). Through transcriptome and biochemical analyses, the defective phenotypes of the (p)ppGpp^0^ strain were traced to a metabolic dysregulation that resulted in toxic accumulation of endogenously produced reactive oxygen species (ROS) and an impaired metal homeostasis (Gaca et al., 2013; Colomer-Winter et al., 2017). In several cases, growth/survival defects of the (p)ppGpp^0^ strain could be rescued by addition of glutathione or manganese supplementation, which either directly or indirectly mitigate ROS damage (Colomer-Winter et al., 2017). In agreement with previous findings obtained with other Firmicutes (Bennett et al., 2007; Geiger et al., 2012; Whiteley et al., 2015), inactivation of *codY* restored the virulence of the (p)ppGpp^0^ strain in the *G. mellonella* and mouse CAUTI models (Colomer-Winter et al., 2017, 2019). Still, the molecular mechanisms by which (p)ppGpp supports virulence in this species cannot be solely attributed to association of (p)ppGpp with the CodY regulon as (i) basal levels of (p)ppGpp that are unlikely to interfere with CodY activity are more relevant to enterococcal pathogenesis than the SR, and (ii) (p)ppGpp controls several genes in a CodY-independent manner during active growth and during the SR (Gaca et al., 2012; Colomer-Winter et al., 2019).

Similar to clinical observations made with *S. aureus* persistent infections (see text above), whole genome sequencing identified a single missense mutation in the Rel_*Efm*_ gene of an antibiotic resistant *E. faecium* that was isolated from a bacteremia case (Honsa et al., 2017). This SNP resulted in constitutively high levels of ppGpp directly linking (p)ppGpp to antibiotic tolerance in *E. faecium*. Interesting, this mutation did not change the minimum inhibitory concentration (MIC) for several antibiotics under *in vitro* conditions, including antibiotics that had been administered to the infected patient (Honsa et al., 2017). Collectively, the picture that emerges is that, similarly to staphylococci, increases in (p)ppGpp basal pools can be directly linked to antibiotic tolerance without necessarily conferring antibiotic resistance.

### Streptococci

The genus *Streptococcus* harbors some of the most commonhuman and animal pathogens. Even though seminal studies that helpedshape our understanding of how (p)ppGpp is metabolized in Gram-positive bacteria were performed with streptococci, from the first insights into the intramolecular regulation of the two catalytic domains of the Rel enzyme (Mechold et al., 2002; Sajish et al., 2007) to the discovery of SASs (Lemos et al., 2007), very few studies directly probed the significance of (p)ppGpp (and the SR) to streptococcal pathogenesis. In *S. pneumoniae*, which only harbors a single SAS (RelQ_*Sp*_), inactivation of the bifunctional *rel*_*Sp*_ supported that Rel_*Sp*_ is the primary source of (p)ppGpp and fully responsible for SR activation (Kazmierczak et al., 2009). Moreover, Rel_*Sp*_ was shown to play a major role in disease progression in a pneumonia mouse model, and was linked to induction of the *ply* operon, which encodes the pneumolysin toxin involved in early infection and tissue invasion (Kazmierczak et al., 2009). However, the exact mechanism by which Rel_*Spn*_/(p)ppGpp regulates this virulence factor remains to be elucidated. In *S. suis*, an important pathogen of pigs, a (p)ppGpp^0^ strain lacking both *rel*_*Ss*_ and *relQ*_*Ss*_ displayed a number of phenotypic defects that can be linked to *S. suis* pathogenesis including decreased capacity to adhere and invade Hep-2 cells, lower survival in whole blood and decreased anti-phagocytic capacity (Zhu et al., 2016). Not surprisingly, virulence of the *S. suis* (p)ppGpp^0^ strain was attenuated in a systemic infection mouse model (Zhu et al., 2016). However, different than *S. aureus*, *E. faecalis*, and *Listeria monocytogenes* (see below), the *S. suis* Δ*rel*Δ*relQ*Δ*codY* triple mutant phenocopied the Δ*rel*Δ*relQ* double mutant in the mouse infection model (Zhu et al., 2019a). Moreover, CodY was shown to interact with the Rel_*Ss*_ promoter in a GTP-independent manner, suggesting a new mechanism of (p)ppGpp and CodY crosstalk.

### Listeria monocytogenes

*Listeria monocytogenes* is an intracellular foodborne pathogen and the causative agent of human listeriosis (Radoshevich and Cossart, 2018). Similar to *S. aureus* and *Streptococcus mutans*, the genome of *L. monocytogenes* encodes a bifunctional Rel_*Lm*_ and the SASs RelP_*Lm*_ and RelQ_*Lm*_. In one of the first studies to provide direct evidence of the association of (p)ppGpp with bacterial virulence, a transposon insertion library identified *rel*_*Lm*_ as an essential gene in a murine model of listeriosis (Taylor et al., 2002). In a study that explored the relationship between (p)ppGpp and CodY, the attenuated virulence of a Δ*rel*_*Lm*_ strain could be partially linked to continued CodY-dependent repression as inactivation of *codY* in the Δ*rel*_*Lm*_ background strain (Δ*rel*_*Lm*_Δ*codY*) partially restored *L. monocytogenes* virulence (Bennett et al., 2007). However, the reduced survival of the Δ*rel*_*Lm*_ strain in culture cell lines was not restored in the Δ*rel*_*Lm*_Δ*codY* strain (Bennett et al., 2007). Follow up studies demonstrated that virulence of a triple mutant strain lacking all three (p)ppGpp synthetases (Δ*rel*_*Lm*_Δ*relP*_*Lm*_Δ*relQ*_*Lm*_) was severely attenuated in an *in vitro* plaque assay (used as a surrogate for *Listeria* virulence), and in a mouse infection model (Whiteley et al., 2015). Similar to what was observed with the single Δ*rel*_*Lm*_ strain (Bennett et al., 2007), inactivation of *codY* partially restored virulence of the (p)ppGpp^0^ strain (Whiteley et al., 2015) collectively suggesting that (p)ppGpp synthesis promotes virulence in *L. monocytogenes* in a CodY-dependent manner. Of note, *L. monocytogenes* was the first bacterial pathogen to identify a regulatory crosstalk between the (p)ppGpp, CodY, and cyclic diadenosine monophosphate (c-di-AMP) signaling pathways (Whiteley et al., 2015).

### Bacillus anthracis

The spore-forming *Bacillus anthracis* is the etiological agent of anthrax (Pilo and Frey, 2018). In *B. anthracis*, activation of the SR, mediated by Rel_*Ba*_, was linked to sporulation but did not affect expression of virulence factors or virulence in a subcutaneous infection mouse model (van Schaik et al., 2007). It should be noted that *B. anthracis* as well as other *Bacillus* species also encode RelP and RelQ (YjbM and YwaC, respectively) such that inactivation of *rel*_*Ba*_ alone can be anticipated to abolish activation of the SR but not basal production of (p)ppGpp. Based on evidence from other Firmicutes, there is a great likelihood that loss of Rel_*Ba*_ hydrolase activity in the presence of active RelP and/or RelQ results in a SR-defective strain with high basal levels of (p)ppGpp. Given the mounting evidence that the association of (p)ppGpp with virulence goes beyond activation of the SR, it will be important to assess the virulence potential of a *B. anthracis* triple Δ*rel*Δ*relP*Δ*relQ* mutant [(p)ppGpp^0^] strain in future investigations.

## Gram-Negative Pathogens

The textbook description of how (p)ppGpp controls bacterial physiology largely derives from studies conducted with the Gram-negative paradigm *E. coli*. In this group of bacteria, (p)ppGpp regulates transcription through direct interaction with the interface of the β′ and ϖ subunits of the RNAP (Ross et al., 2013). Of note, most transcriptional effects triggered by artificially induced early (p)ppGpp accumulation in the absence of stress were recently shown to be caused by (p)ppGpp-RNAP interaction (Sanchez-Vazquez et al., 2019). Moreover, many of the targets subjected to allosteric regulation by (p)ppGpp including proteins involved in translation, DNA replication and purine biosynthesis were fisrt identified in *E. coli* (Dalebroux and Swanson, 2012). In the absence of clinical reports or *in vivo* studies clearly linking (p)ppGpp regulation to *E. coli* virulence, the bulk of our understanding of the importance of (p)ppGpp to the virulence of Gram-negative pathogens derives from studies conducted with other Gammaproteobacteria such as *Pseudomonas aeruginosa, Salmonella enterica*, and *Vibrio cholerae* (Table 1). Different than Firmicutes, (p)ppGpp is metabolized by a monofunctional synthetase (RelA) and a bifunctional synthetase/hydrolase (SpoT) in Gammaproteobacteria (Atkinson et al., 2011). In all organisms with the RelA and SpoT enzyme arrangement, RelA has strong enzymatic activity and is the primary driver of the SR while SpoT has a strong hydrolase activity and weak synthetase activity that is triggered by specific conditions including iron and fatty acid starvation (Vinella et al., 2005; Battesti and Bouveret, 2006). The exception among Gammaproteobacteria, *V. cholerae* encodes a third (p)ppGpp synthetase, which is a SAS-like monofuntional enzyme named RelV (Das et al., 2009). Different than Gammaproteobacteria, Alphaproteobacteria such as *Brucella* sp. and Epsilonproteobacteria such as *Helicobacter pylori* encode a single bifunctional (p)ppGpp synthetase/hydrolase known as Rsh, Rel or SpoT, that seems to be functionally analog to the Gram-positive Rel enzyme as it possess equally strong synthetase and hydrolase activities (Atkinson et al., 2011). While there are numerous studies linking (p)ppGpp to virulence expression in Gram-negative pathogens, many covered in a previous review (Dalebroux et al., 2010), the sections below will focus on recent studies that have directly linked (p)ppGpp to Gram-negative bacterial virulence using animal infection models.

### Salmonella

*Salmonella enterica* is responsible for a variety of human infections ranging from gastroenteritis to typhoid fever. Early studies with (p)ppGpp^0^ strains (Δ*relA*Δ*spoT*) of *S. enterica* serovar Typhimurium, Typhi, and Gallinarum clearly demonstrated the essentiality of (p)ppGpp to *Salmonella* pathogenesis (Pizarro-Cerda and Tedin, 2004; Jeong et al., 2008; Park et al., 2010; Dasgupta et al., 2019). Interestingly, (p)ppGpp was found to regulate the expression of *Salmonella* pathogenicity islands 1 (SPI-1) and 2 (SPI-2), which are required for *Salmonella* virulence (Pizarro-Cerda and Tedin, 2004; Song et al., 2004). Follow up RNA sequencing (RNAseq) analysis revealed that transcription of more than 30% of the *Salmonella* coding regions and approximately 20% of non-coding regions was affected by fluctuations in (p)ppGpp levels (Ramachandran et al., 2012), further confirming the far-reaching scope of (p)ppGpp regulation. To better understand the contributions of RelA and SpoT, a recent study characterized a *spoT* mutant (*spoT*Δctd strain), unable to synthesize (p)ppGpp via SpoT due to the deletion of the *C*-terminal domain (ctd) regulatory element of the enzyme without affectting its hydrolase activity, and revealed that RelA was the primary enzyme responsible for nutrient, nitrosative and oxidative stresses, while (p)ppGpp synthesized by SpoT was important for adaptation and survival within phagocytes (Fitzsimmons et al., 2020). More specifically, the *spoT*Δctd strain failed to induce SPI-2 genes in response to the acidic pH of the phagosome, had a major defect in cation metal uptake, and was highly attenuated in a murine model of acute salmonellosis (Fitzsimmons et al., 2020). Thus, in addition to its well-recognized role in environmental stress adaptation, (p)ppGpp appears to also function as an intracellular signal for *Salmonella* virulence. Finally, single immunization of mice with a live (p)ppGpp^0^ strain elicited both systemic and mucosal antibody responses and protected vaccinated animals from a subsequent challenge with a lethal dose of wild-type *S. typhimurium* (Na et al., 2006). Given the high potential of targeting (p)ppGpp metabolism for the development of anti-infective therapies, additional studies are warranted to determine the distinct roles of RelA, SpoT, and of basal (p)ppGpp pools in *Salmonella*.

### Pseudomonas aeruginosa

This environmental organism is well known for its remarkable ability to form biofilms on different types of surfaces, intrinsic and acquired tolerance to multiple antibiotics, and association with chronic lung infections in cystic fibrosis patients, burn wound infections and serious nosocomial infections (Gellatly and Hancock, 2013). In the absence of RelA, cells treated with the serine analog serine hydroxamate failed to accumulate (p)ppGpp, confirming previous observations that RelA is responsible for activation of the SR during amino acid starvation (Erickson et al., 2004). While virulence of Δ*relA* and Δ*relA*Δ*spoT* [(p)ppGpp^0^] mutant strains was significantly attenuated in a *Drosophila melanogaster* feeding model of infection (Erickson et al., 2004), only the double mutant showed loss of virulence in a rat lung agar bead and in a murine skin infection model (Vogt et al., 2011; Pletzer et al., 2017). Furthermore, basal levels of (p)ppGpp was linked to expression of important virulence determinants such as alginate, type-three secretion system (T3SS), pyocyanin, proteases, siderophores, swarming, twitching and other forms of motility, at least in part due to a crosstalk with the quinolone quorum-sensing system (Vogt et al., 2011). In addition, a *P. aeruginosa* (p)ppGpp^0^ strain showed decreased cytotoxicity toward human alveolar adenocarcinoma cell lines and human epithelial cells, reduced hemolytic activity, impaired virulence in a cutaneous abscess model, and reduced mortality, lung edema and inflammatory cell infiltration in a mouse model of acute pneumonia (Xu et al., 2016). In terms of antibiotic tolerance, (p)ppGpp has been associated to *P. aeruginosa* multidrug tolerance upon stationary phase entry through activation of antioxidant defenses. Specifically, lack of (p)ppGpp led to loss of superoxide dismutase (SOD) activity, while genetic or chemical complementation of SOD activity in the Δ*relA*Δ*spoT* strain restored antibiotic tolerance (Martins et al., 2018).

### Burkholderia pseudomallei

*Burkholderia pseudomallei* (formally *Pseudomonas pseudomallei*) is the causative agent of melioidosis, a disease of both humans and animals, classified by the CDC as a category B select agent (Wiersinga et al., 2018). *Burkholderia* spp. are known to be metabolically versatile, to thrive under adverse conditions and to tolerate antibiotic treatment. A *B. pseudomallei* strain lacking the *relA* and *spoT* genes [(p)ppGpp^0^], displayed defects in stationary-phase survival, replication within macrophages, and attenuated virulence in the *G. mellonella* invertebrate model as well as acute and chronic mouse models of melioidosis (Muller et al., 2012). Similar to *Salmonella*, vaccination of mice with the (p)ppGpp^0^ strain conferred partial protection against subsequent infection with wild-type *B. pseudomallei* (Muller et al., 2012). The distinct roles of RelA and SpoT in the infection process and in antibiotic tolerance, and the extent of the (p)ppGpp regulatory network remain to be explored in *B. pseudomallei* and other pathogenic *Burkholderia* spp.

### Vibrio cholerae

A water-borne pathogen and the causative agent of cholera, *V. cholerae* is a versatile pathogen with important virulence factors such as the cholera toxin (CT) and the toxin co-regulated pilus (TCP) (Childers and Klose, 2007). As indicated above, in addition to the canonical RelA and SpoT, the genome of *V. cholerae* encodes a SAS named RelV that is unique to *Vibrio* species (Das et al., 2009). Early studies linked RelA-dependent (p)ppGpp production with optimal expression of CT, TCP, and two major virulence regulators (ToxR and ToxT), and with virulence in rabbit ileal loop and suckling mouse infection models (Haralalka et al., 2003). However, contradictory findings were subsequently made with a new *relA* mutant that produced normal levels of CT and TCP and displayed no colonization defects in the suckling mouse model (Silva and Benitez, 2006). Later reports using a double Δ*relA*Δ*spoT* mutant strain – an overproducer of basal (p)ppGpp levels due to the activity of RelV – produced higher levels of CT, whereas anaerobic growth via trimethylamine oxide respiration was severely inhibited (Oh et al., 2014). In contrast, a ppGpp^0^ strain (Δ*relA*Δ*spoT*Δ*relV*) grew substantially better, but produced no CT, collectively suggesting that CT production and bacterial growth are inversely regulated in response to (p)ppGpp accumulation (Oh et al., 2014). Moreover, virulence of the Δ*relA*Δ*spoT*Δ*relV* strain was significantly attenuated in the infant suckling mouse model (Oh et al., 2014). More recently, (p)ppGpp was also shown to contribute to antibiotic tolerance of *V. cholerae*, possibly by suppressing TCA cycle activity that lowered ROS production (Kim et al., 2018).

### Acinetobacter baumannii

An opportunistic pathogen, *Acinetobacter baumannii* has emerged as a leading cause of hospital-acquired infections, in large part due to its stress resilience and multidrug tolerance (Harding et al., 2018). A Δ*relA* strain failed to produce detectable levels of ppGpp during amino acid starvation and was hypermotile while showing reduced tolerance to antibiotics and attenuated virulence in the *G. mellonella* model (Perez-Varela et al., 2020). In a separate study, lack of (p)ppGpp resulted in lower expression of several efflux pump genes, providing a possible explanation for the association of (p)ppGpp with antibiotic tolerance in this organism (Jung et al., 2020). Studies to determine the consequences of a complete loss of (p)ppGpp that should only be achieved in a Δ*relA*Δ*spoT* double mutant strain to the pathophysiology of *A. baumannii* are still warranted.

### Haemophilus ducreyi

*Haemophilus ducreyi* causes the sexually transmitted disease chancroid, a major cause of genital ulceration in developing countries (Lewis and Mitjà, 2016). The conditions encountered in human lesions are thought to resemble those found during stationary phase *in vitro* growth and; in line with this, a (p)ppGpp^0^ Δ*relA*Δ*spoT* mutant was attenuated for pustule formation in human volunteers (Holley et al., 2014). However, the (p)ppGpp^0^ strain displayed conflicting phenotypes *in vitro* as it was more sensitive to oxidative stress, but showed increased resistance to phagocytosis and prolonged survival in the stationary phase (Holley et al., 2014). RNAseq analysis of *H. ducreyi* grown to stationary phase indicated that loss of (p)ppGpp resulted in the dysregulation of several of its virulence determinants, including reduced production of Flp adhesin proteins (Holley et al., 2015). More recently, the *H. ducreyi* transcriptome in biopsy specimens of human lesions was compared to bacteria grown to mid-log, transition or stationary phases. While many of the genes previously shown to be regulated by (p)ppGpp were not differentially expressed in this study, genes coding for proteins involved in nutrient transport and alternative carbon utilization pathways, which are typically controlled by (p)ppGpp, were upregulated during infection (Gangaiah et al., 2016). Further characterization of the impact of the SR to *H. ducreyi* virulence and antibiotic tolerance by using *relA* and *spoT* single mutants coupled with cellular (p)ppGpp quantifications might shed new light on these findings.

### Francisella tularensis

*Francisella tularensis* is the causative agent of tularemia, which is transmitted to humans upon contact with infected animals (Jones et al., 2014). Because of its high lethality in its pneumonic form and extremely low infectious dose, *F. tularensis* is classified by the United States Center for Disease Control (CDC) as a Category A select agent. Several studies have implicated (p)ppGpp with virulence gene expression of *F. tularensis*, with the most current model indicating that (p)ppGpp promotes virulence in this pathogen by activating transcription of the *Francisella* pathogenicity island (FPI) through interactions with the DNA-binding protein PigR and the MglA-SspA-RNAP complex (Cuthbert et al., 2017; Rohlfing et al., 2018). Indeed, virulence of a double Δ*relA*Δ*spoT* strain was shown to be attenuated in intranasally infected mice (Ma et al., 2019). Similar to observations made with *P. aeruginosa* and *V. cholerae*, (p)ppGpp was shown to govern global transcriptional changes in response to oxidative stress and required for tolerance to oxidants, most likely supporting intraphagocytic survival (Ma et al., 2019). In the closely related *F. novocida* – a laboratory surrogate of *F. tularensis* – survival of a *relA* mutant was impaired in the J774. A macrophage cell line, and its virulence was attenuated in the mouse model of tularemia (Dean et al., 2009). To date, *relA* and *spoT* single mutants of *F. tularensis* have not been characterized and it is unknown how loss of RelA-mediated SR (rather than complete absence of (p)ppGpp of a *relA spoT* double mutant strain) will impact *F. tularensis* virulence. Similar to *Salmonella* and *Burkholderia* (p)ppGpp-deficient strains, infection with the Δ*relA* mutant elicited a protective immune response in mice, further supporting the potential of (p)ppGpp-deficient strains as attenuated live vaccines (Dean et al., 2009).

### Brucella

The intracellular pathogen *Brucella melitensis*, the causative agent of brucellosis, possesses a single (p)ppGpp synthetase/hydrolase enzyme known as *rsh*. Inactivation of *rsh* resulted in altered morphology, reduced intracellular growth/survival in HeLa and ovine macrophages, and attenuated virulence in a mouse infection model (Dozot et al., 2006). The attenuated virulence of the (p)ppGpp^0^ strain was attributed, at least in part, to (p)ppGpp controlling the expression of the type four secretion system (T4SS) VirB, a major virulence factor of Brucellae (Dozot et al., 2006).

## Other Bacteria

### Mycobacterium

A distinguishing characteristic of *Mycobacterium* species is the presence of a hydrophobic mycolate layer attached to the peptidoglycan by an intermediate arabinogalactan layer. The most relevant member of this genus is *M. tuberculosis*, which causes tuberculosis in humans, while some other species are opportunistic animal and human pathogens. The genome of *M. tuberculosis* encodes a bifunctional Rel enzyme and a SAS enzyme which is phylogenetically distinct from the RelP and RelQ enzymes of Firmicutes and the *V. cholerae* RelV (Atkinson et al., 2011).

In *M. tuberculosis*, (p)ppGpp regulates multiple phenotypes, including biofilm formation, latency and antibiotic tolerance (Primm et al., 2000; Dahl et al., 2003; Weiss and Stallings, 2013). Similar to Firmicutes, activation of the SR in *M. tuberculosis* is primarily mediated by Rel_*Mtb*_. Inactivation of *rel*_*Mtb*_ significantly attenuated *M. tuberculosis* virulence in a mouse model of chronic lung infection (Dahl et al., 2003) and in a guinea pig lung infection model (Klinkenberg et al., 2010). Notably, the pathology of guinea pig lungs infected with the *rel*_*Mtb*_ mutant was markedly different showing a delayed hypersensitive response. Collectively, these studies indicate that the SR is not required for initial colonization and growth in the lungs, but essential for chronic infection. In a separate study, a strain harboring a point mutation in *rel*_*Mtb*_ that silenced its synthetase activity without disrupting the hydrolase activity phenocopied the *rel*_*Mtb*_ deletion strain, as it failed to persist in the lungs of infected mice (Weiss and Stallings, 2013). A double mutant strain lacking both *rel*_*Mtb*_ and the SAS-encoding gene, presumably a (p)ppGpp^0^ strain, also phenocopied the *rel*_*Mtb*_ single mutant in this mouse model (Weiss and Stallings, 2013), suggesting that the SR might be more relevant to *M. tuberculosis* pathophysiology than basal levels of (p)ppGpp. In addition to playing an essential role in the latency stage, likely through regulation of central metabolism, transcriptional studies indicate that (p)ppGpp also controls the expression of virulence genes. Specifically, the expression of several polyketide synthases that function as immune modulators and surface proteins important for granuloma formation are regulated in a Rel_*Mtb*_-dependent manner (Dahl et al., 2003).

### Borrelia burgdorferi

The etiological agent of Lyme disease is a tick-borne obligate intracellular pathogen and a member of the phylum Spirochetes (Steere et al., 2016). The zoonotic life cycle of *Borrelia burgdorferi* involves adapting to a variety of stresses, including nutrient starvation in arthropod hosts. In addition, *B. burgdorferi* must tolerate adverse conditions during infection of the human host. In Spirochetes, a single bifunctional Rel_*Bbu*_ enzyme (also known as SpoT) is responsible for (p)ppGpp metabolism. Despite conflicting results on whether (p)ppGpp levels increase during conditions that mimic the nutrient-limiting conditions encountered by *B. burgdorferi in vivo* (Bugrysheva et al., 2003; Drecktrah et al., 2015), *rel*_*Bbu*_ was required for full virulence in an intradermal infection mouse model (Bugrysheva et al., 2005). Subsequent studies revealed that (p)ppGpp modulates glycerol uptake and utilization, the morphological conversion that occurs during nutrient starvation, and persistence in the tick (Bugrysheva et al., 2015; Drecktrah et al., 2015).

## ppGpp Signaling as a Therapeutic Target

As the current literature strongly supports that (p)ppGpp is critical for bacterial fitness, virulence and antibiotic tolerance, the identification of molecules that interfere with (p)ppGpp signaling has been actively pursued by investigators around the globe. The first antibiotic to be associated with decreases in alarmone levels was chloramphenicol (Rodionov and Ishiguro, 1995), which was later confirmed and expanded to include other protein synthesis inhibitors (Kudrin et al., 2017). However, the effects of these antibiotics to (p)ppGpp metabolism and activation of the SR were not specific. To date, the lead compounds identified as ppGpp and/or SR inhibitors are classified into two groups: (i) molecules that directly inhibit (p)ppGpp synthesis by interfering with the activity of (p)ppGpp synthetases, and (ii) molecules that promote (p)ppGpp degradation. In the group of (p)ppGpp synthesis inhibitors, the first identified compound was relacin, a synthetic (p)ppGpp analog based on the crystal structure of the *S. equisimilis* Rel (Rel_*Seq*_) enzyme (Wexselblatt et al., 2012). While shown to inhibit (p)ppGpp synthetic activity of both the Gram-negative RelA and Gram-positive Rel enzymes *in vitro* (Wexselblatt et al., 2012), relacin does not penetrate the intracellular compartment of Gram-negative bacteria. Thus, the antimicrobial properties of relacin was restricted to Gram-positive pathogens as it was shown to impair cell survival, biofilm formation of *S. pyogenes* and *B. anthracis* and sporulation of *B. anthracis* (Wexselblatt et al., 2012). Based on these promising results, follow up studies modified the relacin structure to develop more potent inhibitors, with two new relacin analogs shown to effectively lower intracellular (p)ppGpp and impair biofilm formation and survival of *Myocobacterium smegmatis* and biofilm formation of *M. tuberculosis* (Wexselblatt et al., 2013; Syal et al., 2017b). Importantly, these compounds were shown to be permeable and non-toxic to human cells (Syal et al., 2017b). However, even the most effective relacin analog showed inhibitory effects in the millimolar range such that further improvements to achieve inhibition in the nanomolar range are deemed necessary prior to testing in human subjects. It should be noted that relacin does not inhibit the activity of the purified *E. faecalis* RelQ enzyme (Gaca et al., 2015b), such that production of (p)ppGpp will most likely not be completely abolished in organisms that encode SASs when treated with relacin or its current analogs.

In addition to relacin, other compounds have been shown to inhibit alarmone synthesis, albeit with low specificity and at even higher concentrations. For example, vitamin C inhibited Rel-dependent (p)ppGpp production in *M. smegmatis* decreasing its long term survival and biofilm formation capacities (Syal et al., 2017a). In another study, a high-throughput screening assay of a library containing ∼2 million compounds against a recombinant Rel_*Mtb*_ identified one compound with Rel_*Mtb*_-specific inhibitory activity that showed synergy with isoniazide in the treatment of *M. tuberculosis* in a mouse lung infection model (Dutta et al., 2019).

As mentioned above, another useful strategy to interfere with (p)ppGpp signaling is to promote (p)ppGpp hydrolysis rather than interfere with its synthesis. Along this line of thought, the anti-biofilm activity of peptide 1018, a synthetic peptide based on the host defense protein bactenecin, was linked to increased (p)ppGpp degradation (de la Fuente-Nunez et al., 2014) albeit the specific association of peptide 1018 with (p)ppGpp has been challenged with its activity suggested to derive from its general physicochemical properties (Andresen et al., 2016a). Apart from the controversial interpretation of the antimicrobial properties of peptide 1018, the synthetically modified DJK-5 and DJK-6 analogs were found to be more potent than peptide 1018 and to confer protection against *P. aeruginosa* infection in two invertebrate models (de la Fuente-Nunez et al., 2015). Taking a step further, DJK-5 was shown to reduce tissue damage and lesion size caused by either *S. aureus* or *P. aeruginosa* in a murine cutaneous abscess model (Mansour et al., 2016). Finally, this same group showed that *P. aeruginosa spoT* promoter activity was suppressed by treatment with peptides DJK-5 and 1018, and that a peptide-treated *relA* complemented SR double mutant strain exhibited reduced peptide susceptibility in the murine subcutaneous abscess model (Pletzer et al., 2017).

To date, several high-throughput screening assays are in place (Andresen et al., 2016b; Beljantseva et al., 2017) and compounds such as relacin can provide proof-of-principle evidence of the potential of (p)ppGpp signaling as the target for antimicrobial drug development. However, more studies are needed before (p)ppGpp signaling inhibitors can be tested in the clinical setting. For instance, even if more potent compounds are identified, additional studies will be necessary to assess their toxicity to humans, biodistribution and pharmacokinetics. Initially thought to be absent in eukaryotes, studies conducted in the past decade have identified SpoT orthologs in plants, insects, and humans (Sun et al., 2010; Tozawa and Nomura, 2011). While the insect and human genes appear to code for the (p)ppGpp hydrolytic domain with no evidence of functioning as a syhthetase, inactivation of the *D. melanogaster spoT* ortholog led to phenotypes that resemble those found in (p)ppGpp-deficient bacteria (Sun et al., 2010). In addition, one must also take into consideration that the significance of (p)ppGpp signaling and the enzymes responsible for (p)ppGpp metabolism may vary among bacterial groups. Specifically, while in some cases persistence and virulence can be associated with activation of the SR (mediated by RelA/Rel enzymes), in other cases basal (p)ppGpp pools during active growth and below the levels needed to activate the SR, appear to mediate those phenotypes. Thus, the ideal (p)ppGpp inhibitor must be capable of inhibiting the enzymatic activity of the so-called long RSHs (RelA, SpoT, Rel) and of SASs (RelP, RelQ, RelV). Alternatively, an effective antimicrobial might function by tipping the balance of bifunctional enzymes toward (p)ppGpp degradation. Apart from these challenges, the development of antibacterial strategies that target (p)ppGpp signaling have several advantages when compared to the antibiotics that are currently available. First, as non-essential enzymes, (p)ppGpp inhibitors will interfere with bacterial fitness and virulence expression but not cell viability such that drug resistance mechanisms may not arise rapidly or may not be acquired at all. Second, perhaps the most promising strategy, considering accumulating evidence that (p)ppGpp mediates bacterial persistence, (p)ppGpp inhibitors could be used in combination with currently available antibiotics that depend on actively growing cells to be effective.

## Concluding Remarks

In addition to the expression of classic virulence factors such as toxins, capsule and fimbriae, contemporary investigations into the mechanisms of bacterial pathogenesis revealed that core cellular processes associated with metabolism and stress tolerance can be equally or even more critical to bacterial pathogenesis. As a global stress regulator, (p)ppGpp signaling appears to provide bacteria with an “extra edge,” increasing cell fitness by controlling central metabolism adjustments in response to environmental fluctuations, activating stress responses, and coordinating expression of classic virulence factors (Figure 2). Importantly, the regulatory effects of (p)ppGpp that initially were thought to be linked to the SR activation is now known to occur in an incremental manner as opposed to the on/off switch that is characteristic of the SR (Gaca et al., 2015a). The picture that emerges from recent studies is that the role of either the SR or basal levels of (p)ppGpp to bacterial virulence depends on the lifestyle and metabolic versatility of each organism.

Despite much progress made in recent years, there are still several aspects of (p)ppGpp regulation that are not well understood. In this regard, the relatively recent discovery of a crosstalk between the (p)ppGpp and c-di-AMP signaling networks may provide new clues. c-di-AMP is an essential signaling nucleotide reported to regulate a variety of cellular functions, in particular osmoregulation (Stulke and Kruger, 2020). Parallel studies conducted with *B. subtilis*, *L. monocytogenes*, and *S. aureus* identified a link between the c-di-AMP and (p)ppGpp signaling pathways (Rao et al., 2010; Corrigan et al., 2015; Huynh et al., 2015). It follows that the phosphodiesterases that are responsible for c-di-AMP degradation are subject to allosteric inhibition by ppGpp, such that high levels of (p)ppGpp correlate with high levels of c-di-AMP; studies with *S. aureus* have also shown an overlap between the c-di-AMP and SR transcriptional signatures (Corrigan et al., 2015; Huynh et al., 2015). In *L. monocytogenes*, a diadenylate cyclase (*dacA*) mutant harbored suppressor mutations in the synthetase domain of the bifunctional Rel enzyme, which led to reduced (p)ppGpp levels (Whiteley et al., 2015). Mutational analysis confirmed that *dacA* was essential in wild-type but not in a (p)ppGpp^0^ strain (Whiteley et al., 2015). Further studies revealed that c-di-AMP was essential because accumulated (p)ppGpp altered GTP concentrations, thereby affecting CodY activity (Whiteley et al., 2015). While the details of the relationship between c-di-AMP and the (p)ppGpp-CodY networks are not well understood, one can hypothesize that (p)ppGpp acts in concert with c-di-AMP to regulate bacterial activities important for adaptation to new environments. Therefore, a better understanding of the c-di-AMP regulatory mechanisms and identification of its targets may fill some of the gaps in our current understanding of how (p)ppGpp promote cell fitness and virulence.

## Author Contributions

SK, CC-W, and JL wrote and reviewed the manuscript. All authors contributed to the article and approved the submitted version.

## Conflict of Interest

The authors declare that the research was conducted in the absence of any commercial or financial relationships that could be construed as a potential conflict of interest.
